# High proportion of PD-1 and CD39 positive CD8+ tissue resident T lymphocytes correlates with better clinical outcome in resected human oesophageal adenocarcinoma

**DOI:** 10.1007/s00262-024-03799-y

**Published:** 2024-09-05

**Authors:** Samuel L. Hill, Gessa Sugiyarto, Jack Harrington, Edward James, Timothy J. Underwood, Tim Elliott

**Affiliations:** 1 School of Cancer Sciences, Faculty of Medicine, University of Southampton, Southampton, UK; 2 Oxford Cancer Centre for Immuno-Oncology and CAMS-Oxford Institute, Nuffield Department of Medicine, University of Oxford, Oxford, UK

**Keywords:** Oesophageal cancer, Adenocarcinoma, CD8+ Lymphocyte, Tissue resident memory cell, Neoadjuvant therapy

## Abstract

**Objective:**

To understand the CD8+ tumour infiltrating lymphocyte (TIL) compartment of oesophageal adenocarcinoma (OAC) with regards to markers of lymphocyte exhaustion, tissue residency and to identify possible reasons behind differential responses to therapy.

**Design:**

Tumour samples from 44 patients undergoing curative resection for OAC were assessed by flow cytometry for presence of antigen-experienced TILs and markers of activation and exhaustion. Populations of PD-1 and CD39 positive OAC TILs were sorted, and bulk RNA sequencing undertaken using a modified SmartSeq2 protocol. Flow cytometric assessment of functionality was completed.

**Results:**

A higher proportion of antigen experienced CD8+ OAC TILs was associated with improved survival following surgery; while, high double positivity (DP) for PD-1 and CD39 among these TILs also correlated significantly with outcome. These DP TILs possess a minority population which is positive for the markers of exhaustion TIM3 and LAG3. Transcriptomic assessment of the PD-1 and CD39 DP TILs demonstrated enrichment for a tissue resident memory T lymphocyte (TRM) phenotype associated with improved survival in other cancers, reinforced by positivity for the canonical TRM marker CD103 by flow cytometry. This population demonstrated maintained functional capacity both in their transcriptomic profile, and on flow cytometric assessment, as well as preserved proliferative capacity.

**Conclusion:**

Resected OAC are variably infiltrated by PD-1 and CD39 DP TILs, an abundance of which among lymphocytes is associated with improved survival. This DP population has an increased, but still modest, frequency of TIM3 and LAG3 positivity compared to DN, and is in keeping with a functionally competent TRM phenotype.

**Supplementary Information:**

The online version contains supplementary material available at 10.1007/s00262-024-03799-y.

## Introduction

Oesophageal adenocarcinoma (OAC) is a lethal disease where less than 20 percent of patients are alive five years after diagnosis. With a minority of patients candidates for curative treatment, and more than half on a radical pathway experiencing disease recurrence, better systemic therapies are needed [1–3]. Despite the propensity for genomic instability and a high tumour mutational burden, the benefits of immune checkpoint blockade (ICB) in oesophageal cancer remain modest with most experiencing minimal benefit, even when biomarker selected [1].

CD8 + tumour infiltrating lymphocyte (TIL) infiltration may impact outcome following surgery in OAC [4]. Among the varied TIL populations, an abundance of tissue resident memory cells (TRM) is associated with responses to ICB in malignant melanoma and non-small cell lung cancer (NSCLC), as well as improved survival post-surgery [5, 6].

A functionally active subset of CD8 and CD4 positive memory T lymphocytes that are restricted to the peripheral tissues, TRM can initiate a rapid response on encountering antigen and can be rapidly recruited and expanded to counter evolving dangers such as viral infections or malignant transformation [7]. Observed across tissue types, where their specific features vary according to their location, TRM possess a core transcriptional profile [8]. They typically express cellular adhesion molecules, particularly the αE integrin CD103, which plays a role in peripheral retention [9]. Additionally, tumour associated TRM express surface markers of lymphocyte exhaustion such as PD-1, TIM3 and CD39 [6, 10]. Understanding the OAC CD8+ TIL compartment allows insight into the composition of the individual tumour immune microenvironment and what may be expected from treatment with ICB.

## Methods

### Samples and disaggregation

All tumour samples were obtained at the time of surgery and accessed via an 8mm punch biopsy from resection specimens. Patients had received either chemotherapy, chemoradiotherapy or no treatment prior to surgery and sample collection (Table S1). All patients were enrolled in the Upper Gastro Intestinal Tumour ecology study at the University of Southampton (REC number 18/NE/0234) with prospective written consent obtained.

Tumour samples were disaggregated into a single cell suspension for either immediate use or storage [11] utilising a combination of mechanical and enzymatic disaggregation with collagenase P (3IU/mL) and DNAse (40IU/mL).

### Flow cytometry

All antibodies used for flow cytometry were titrated for optimal concentrations (Supp Table 1). Extracellular staining was performed in 100µL ice cold MACS buffer. Intracellular staining was performed following incubation in fixation/permeabilization solution 200µL (Foxp3Transcription Factor Staining Buffer Set, Thermo Fisher Scientific) and then stained with antibodies added to 100µL of Permeabilization Wash Buffer. Samples were analysed using Beckton Dickinson (BD) FACSCanto II flow cytometer or a BD LSRFortessa flow cytometer. Data were analysed using FlowJo version 10.8.1 software (FlowJo LLC, BD).

### Lymphocyte isolation

Fluorescence-activated cell sorting (FACS) was undertaken following extracellular staining. Lymphocyte populations sorted using BD FACSAria IIb machine into sterile nuclease free tubes containing 500µL of TRIzol™ solution (Zymo Research), and frozen immediately at − 80°C.

### RNA extraction, cDNA production and sequencing

RNA extraction from sorted cells stored in TRIzol™ solution was undertaken in a dedicated room and hood kept sterile for work using nucleic acid and following sterilisation with UV light and RNase decontamination solution. RNA extraction using Direct-Zol RNA Microprep Kit (Zymo) spin columns was performed as per manufacturers’ protocol. RNA samples were eluted into 15µL nuclease free water and analysed and/or frozen at -80˚C immediately on completion of extraction.

2.5µL of eluted RNA in nuclease free water was used as input for cDNA production following the SmartSeq2 protocol [12]. All primers and reagents as per the original protocol. cDNA processed for tagmentation and indexing in accordance with Nextera XT protocol (Illumina) and multiplexed libraries were sequenced using the NextSeq550 platform (Illumina).

### Statistical analysis

Post sequencing processing included FASTQ alignment to human genome (hg38) using STAR 2.7.6a package. Reads per gene from all samples were transferred to R version 4.0.2 for downstream analysis. Differential gene expression analysis and read count normalisation was undertaken using the DESeq2 package version 1.28.1 for R to produce normalised gene expression counts. Gene Set Enrichment Analysis (GSEA) using the Broad Institute software version 4.1.0 and Gene Set Variation Analysis (GSVA) completed in R using the GSVA (version 1.45.0) package.

Flow cytometry results analysed using FlowJo version 10.8.1 software (FlowJo LLC, BD). Further analysis, statistical significance assessment and figure production in GraphPad Prism version 9.4.1 (Dotmatics). Calculation of p values for flow cytometry data calculated using paired or unpaired t test and survival statistics using Log-rank Mantel-Cox test.

### Functional assessment

Stimulation assays were performed on thawed disaggregated tumour samples, and healthy donor PBMCs. For assessment of degranulation, samples were incubated with 5µL of anti-CD107a antibody with or without Phorbol 12-myristate-13-acetate (PMA) (50ng/mL) and Ionomycin (1ug/mL). Assessment of effector molecule production by CD8+ populations of interest was performed by flow cytometry following incubation at 37°C for 5–6 h, with Brefeldin A (Golgi-Plug™, BD) added at a concentration of 0.1% after 1 h.

## Results

### Lymphocyte infiltration in OAC

**Fig. 1:**
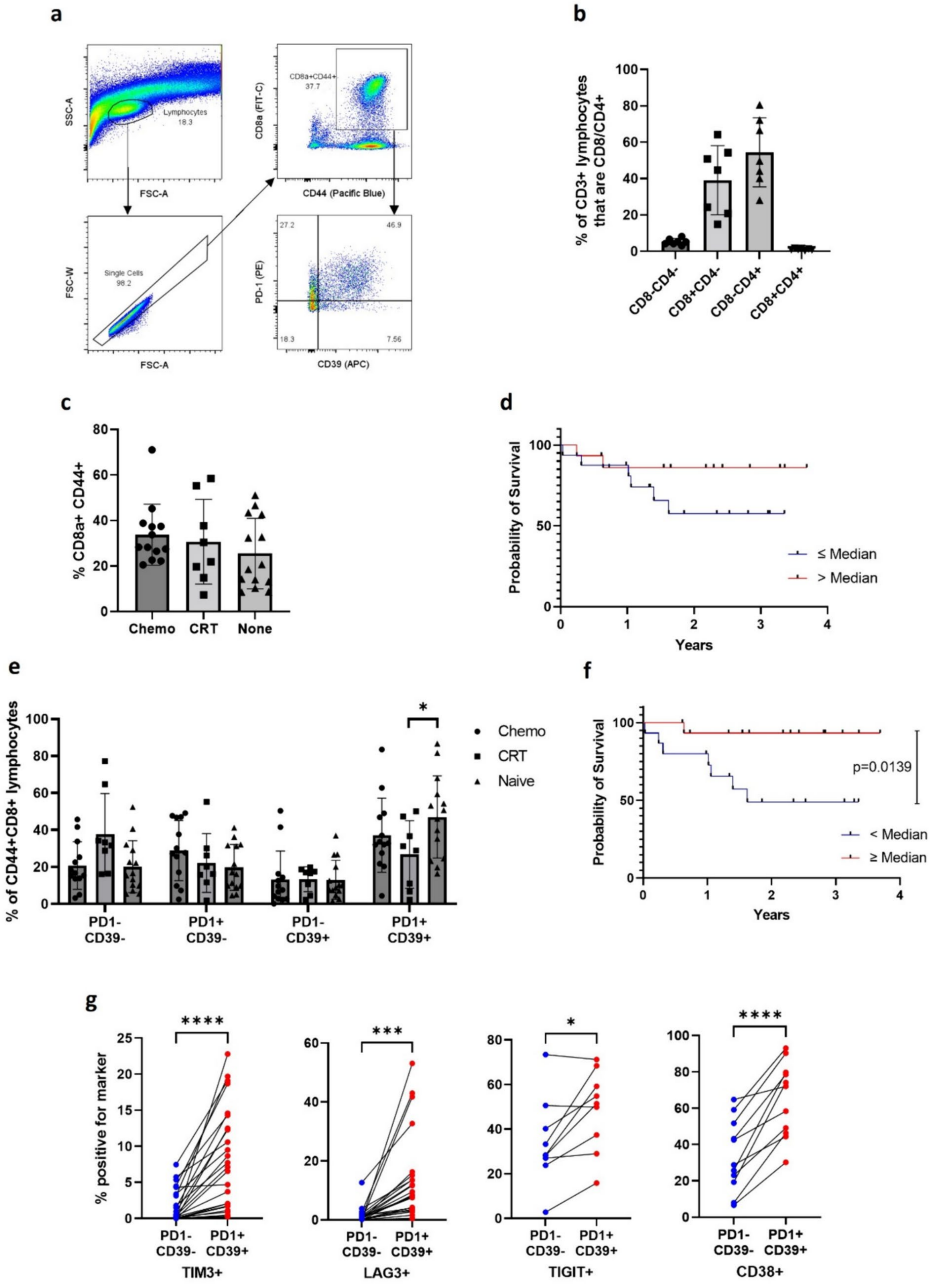
Flow cytometry assessment of resected OAC samples. (**a**) Gating strategy for flow cytometry assessment of disaggregated OAC tumour samples with lymphocytes gated morphologically by forward and side-scatter, cell singlets identified, and antigen experienced CD8+ TILs identified by CD8a and CD44 positivity. This CD8+ TIL population was assessed for PD-1 and CD39, identifying clear double positive population, a high proportion of which correlated with outcome following surgery. Full details of antibodies used available in supplementary table. (**b**) A subset of samples was analysed by flow cytometry for CD3, CD4 and CD8a positivity to assess the gating strategy. Displayed are the percentage of cells identified as lymphocytes morphologically and based upon CD3 positivity, that are positive for CD4 and CD8. Mean and standard deviation shown. **(c)** Percentage of morphologically defined lymphocytes positive for CD8a and CD44 within resected tumours either pre-treated with neoadjuvant chemotherapy, chemoradiotherapy or treatment naïve. (**d**) Improved survival in tumours that possessed a percentage of lymphocytes greater than the median that were positive for CD8a and CD44 (2 year overall survival > median vs ≤ median (86.2% vs 57.5%)). (**e**) Proportion of CD44+ CD8a+ lymphocytes from OAC resection specimens that are PD-1-/CD39-, PD-1+ /CD39-, PD-1-/CD39+ and PD-1+ /CD39+ within the indicated treatment groups (Post neoadjuvant chemotherapy, chemoradiotherapy or treatment naïve) as defined by flow cytometry. Treatment naïve resected specimens had a higher percentage of antigen experienced TILs that were positive for PD-1 and CD39 (47.1%) compared to those treated with chemoradiotherapy (26.8%, *p* = 0.0407, unpaired t test) or neoadjuvant chemotherapy (37.2%, ns). (**f**) Overall survival was significantly higher in patients with a high proportion of OAC TILs positive for PD-1 and CD39 (2 year overall survival ≥ median vs < median (93.3% vs 49.1%) *p* = 0.0139 Log-rank Mantel-Cox test. (**g**) PD-1 and CD39 double positive antigen experienced CD8+ TILs demonstrated increased TIM3, LAG3, TIGIT and CD38 positivity when compared to PD-1 and CD39 double negative populations. Comparisons made using paired t test. * denotes *p* value < 0.05, ** < 0.01, *** < 0.001 and **** < 0.0001

We adopted flow cytometry to characterise CD8+ TILs in resected OAC and captured a mean of 97.8% CD3+ lymphocytes (range 94.5–99.6, *n* = 7) using morphology-based gating alone (Fig. 1a, b). Improved survival was seen in patients with a higher percentage of antigen experience (CD44 +) CD8+ TILs (CD8a+ /CD44+  > median 86.2% OS vs 57.6%, n = 3*1*) (Fig. 1c, d), and co-expression of CD39 and PD-1 was seen in a large fraction of these lymphocytes (mean 38.4% *n* = 35)(Fig. 1e).

An increased proportion of PD-1 and CD39 double positive (DP) TILs among CD44 + CD8+ lymphocytes as measured by flow cytometry, correlated with improved progression free and overall survival (%DP as proportion of total lymphocytes ≥ median, OS *p* = 0.0194) (Fig. 1f). This was notable given the high proportion of DP TILs in some treatment naïve patients (mean 47.1% *n* = 14) who had not received optimal neoadjuvant therapy and in whom poorer outcomes may be expected.

### Flow cytometry assessment of the PD-1+ CD39+ (DP) population

TIM3, LAG3, TIGIT, and CD38 staining was increased in the DP group compared to DN (Fig. 1g); while, TIM3, LAG3, TIGIT and CD39 were all maximally expressed among TILs with high PD-1 expression (Fig. S1a-b). This co-expression of various exhaustion markers with increasing levels of PD-1 has been seen in other cancers including in NSCLC, where PD-1 high CD8+ TILs represent a distinct population enriched for effector functions, tumour reactivity and are associated with response to ICB [13]. Such observations have yet to be demonstrated in OAC, where we demonstrate a relatively low overall abundance of TIM3 and LAG3 positive antigen experienced CD8+ TILs (Fig. 1g).

### Transcriptomic analysis

**Fig. 2:**
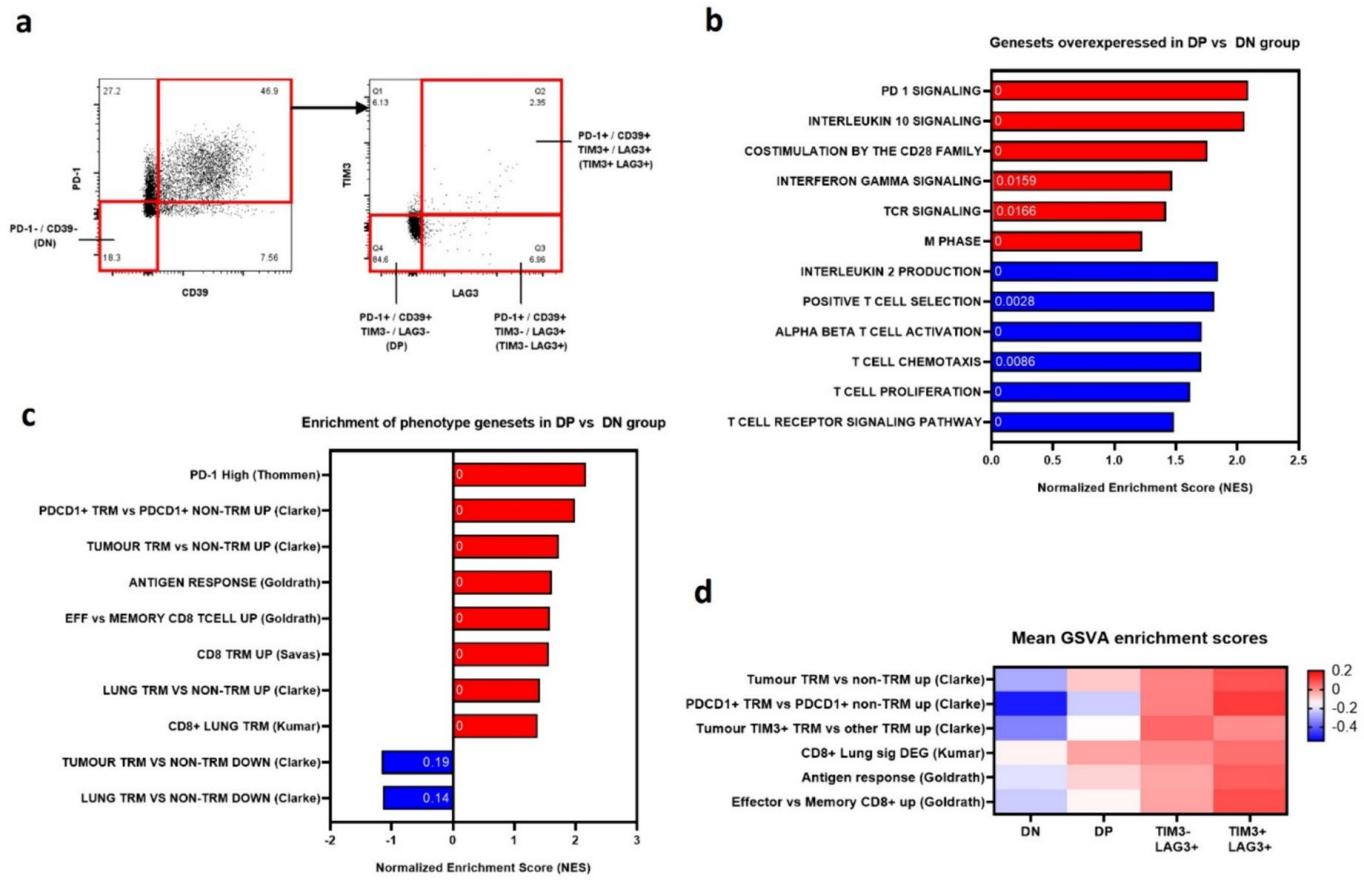
Transcriptomic assessment of PD-1 and CD39 positive populations of antigen experienced CD8+ TILs. (**a**) Populations of CD8a+ CD44+ TILs were isolated using FACS sorting, separating PD-1 and CD39 double negative (DN), and PD-1 and CD39 double positive groups that were either TIM3- LAG3- (double positive / DP), TIM3- LAG3+ , or TIM3+ LAG3+ . (**b**) Normalised enrichment scores (NES) for Reactome (red) and Gene Ontology (blue) signatures in DP vs DN populations, as assessed by gene set enrichment analysis (GSEA). P values for GSEA of each gene set labelled within the bar in white. *P* value of 0 when < 0.00001. (**c**) NES and p values for GSEA analysis of DP vs DN population, using gene sets from canonical descriptions of memory lymphocyte populations (Goldrath) [16], TRM from lung cancer (Thommen, Clarke, Kumar) [6, 8, 13], and breast cancer (Savas) [18]. (**d**) Gene set variation analysis assessing enrichment of each population for key genesets describing memory lymphocyte populations (Goldrath) [16], and TRM from non-small cell lung cancer (Clarke, Kumar) [6, 8, 13]. Heatmap displays mean enrichment score for each population

To characterise these populations further transcriptomic profiles were generated for antigen experienced CD8+ TILs that were DN (*n* = 7, 5 treatment naïve, 2 chemotherapy), DP (*n* = 9, 6 naïve, 3 chemo) as well as for TILs considered likely to represent increasingly exhausted or dysfunctional lymphocytes. This included PD-1 and CD39 positive TILs that were TIM3- but LAG3 + (TIM3-LAG3 +) (*n* = 7, 5 naïve, 2 chemo) or TIM3 and LAG3 positive (TIM3+ LAG3+) (*n* = 6, 4 naïve, 2 chemo) (Fig. 2a). With a high degree of overlap of differentially expressed genes (DEGs) between treatment naïve and chemotherapy pre-treated samples, both groups were combined for analysis.

### Comparison of PD-1-CD39- (DN) and PD1+ CD39+ (DP) tumour infiltrating T cells

Comparing the DP with DN transcriptomes revealed 485 significantly differentially expressed genes (DEGs) (p < 0.05). Among the 66 genes upregulated in the DP group (Supplementary table) were genes observed in lymphocyte exhaustion as well as tissue residency, including *PDCD1* (PD-1), *CD200R1, TNFSF4* (OX40-ligand), *TNFRSF18* (GITR) and *CXCL13* [6, 14]. The latter, a chemoattractant for B lymphocytes, plays an important role in tertiary lymphoid structure formation, and is expressed in conjunction with exhaustion markers in tumour reactive CD8+ TILs [13]. DN TILs showed relative increased expression of numerous immune active molecules with no strong identifying signature indicating some heterogeneity in this population.

An unselected GSEA-based approach assessing Reactome pathways and Gene Ontology (GO) signatures revealed enrichment in the DP group for T cell receptor (TCR), PD-1 and CD28 family signalling, T cell activation and chemotaxis, mitosis, and cell proliferation as well as IL-2 production (Fig. 2b). Less clear trends were observed for the DN group suggesting a heterogeneous population with a difficult to characterise phenotype, an observation made in similar studies. GSEA exploring gene sets relating to lymphocyte differentiation, dysfunction, and exhaustion, showed no DP enrichment for gene sets describing classical lymphocyte exhaustion [15] but strong enrichment for an effector-memory phenotype [16, 17]. This corresponded with TRM populations previously characterised in breast cancer [18] and more strikingly in non-small cell lung cancer [6, 8] (Fig. 2c).

### Comparison of PD-1+ CD39+ TIM3-LAG3- (DP) with PD-1+ CD39+ TIM3-LAG3+ (TIM3-LAG3+) and PD-1 + CD39+ TIM3+ LAG3+ (TIM3+ LAG3+) tumour infiltrating T cells

GO and Reactome pathway analysis was used to compare the DP and TIM3+ LAG3+ groups, with the latter significantly enriched for gene sets associated with mitosis, cell cycle progression, and TCR signalling (Fig. S2a). This, along with a lack of enrichment of canonical LCMV related exhaustion signatures may suggest a functionally active TIL population experiencing persistent TCR signalling. This TIM3+ LAG3+ group also enriched for TRM gene sets, particularly those derived from TIM3+ TRM populations [6], as well as regulation and expression of RUNX3, a key transcription factor driving tissue residency of CD8+ T lymphocytes.

To allow comparison of multiple phenotypes, mean GSVA enrichment scores were generated. This showed increasing PD-1 and TCR signalling from negative values of the DN group, through the DP to maximum values for the TIM3-LAG3+ and TIM3+ LAG3+ groups (Fig. 2d). Additionally, enrichment for gene sets related to TRM were greatest in the TIM3+ LAG3+ groups, decreasing through the TIM3-LAG3+ and DP groups with a negative mean enrichment score for the DN group (Fig. 2d).

### TRM confirmation by CD103 positivity by flow cytometry

**Fig. 3:**
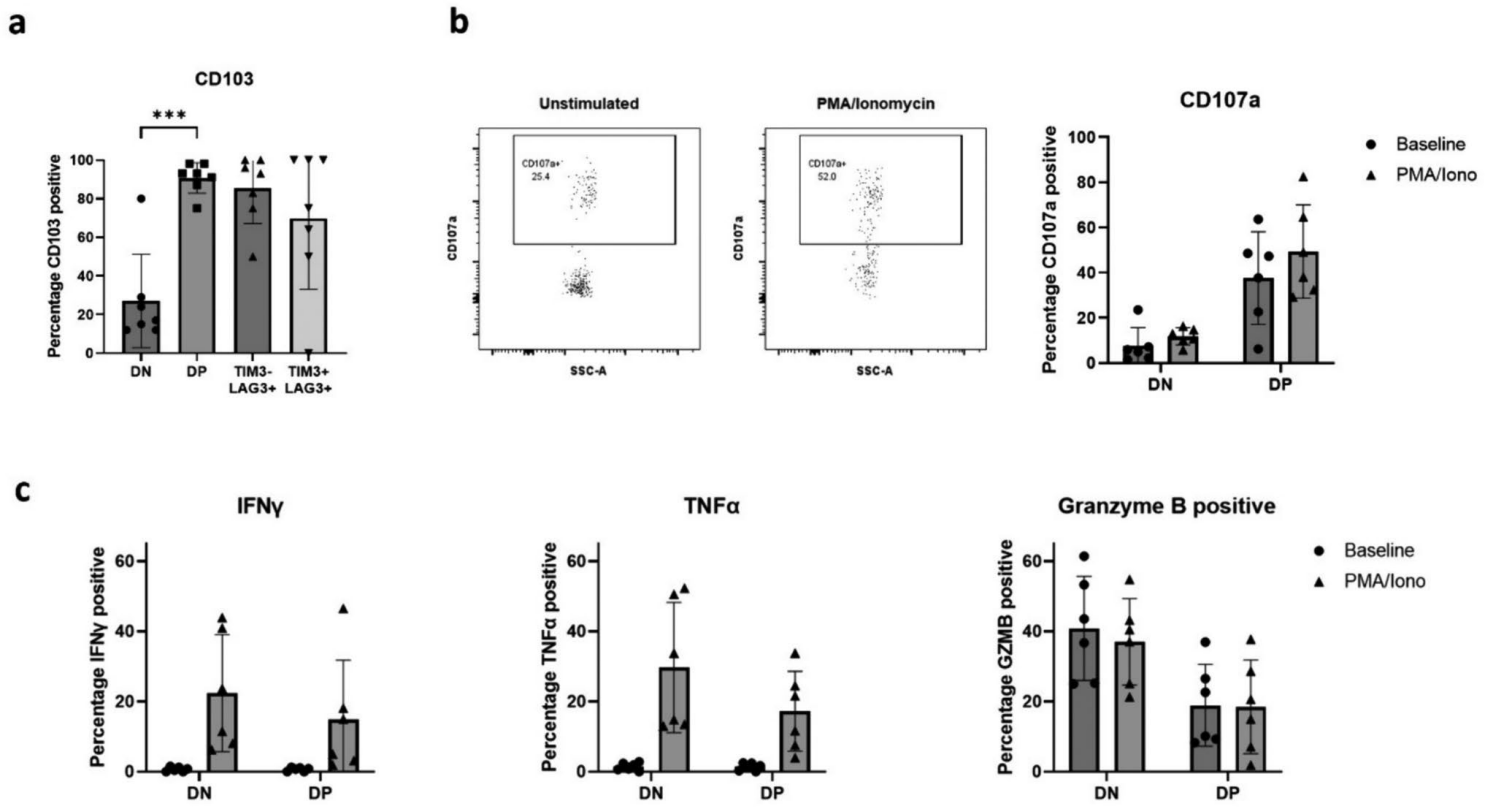
Functional assessment of PD-1 and CD39 positive antigen experienced OAC CD8+ TILs. (**a**) CD103 staining of PD-1- CD39- TIM3- LAG3- double negative (DN), PD-1+ CD39+ TIM3- LAG3- (DP), PD-1+ CD39+ TIM3-LAG3+ (TIM3-LAG3+) and PD-1+ CD39+ TIM3+ LAG3+ (TIM3+ LAG3+) groups (*n* = 7). Statistically significant increase in CD103 positivity in DP vs DN groups, paired t test, *p* = 0.0007. (**b**) Flow cytometry assessment of CD107a positivity, indicating degranulation. CD107a expression at both baseline and on incubation with PMA and Ionomycin (*n* = 6). (**c**) Intracellular staining for IFNγ, TNFα and Granzyme B in PD-1 and CD39 DN and DP groups both at baseline and on incubation with PMA and Ionomycin (*n* = 6)

Confirmation of a TRM population was undertaking by flow cytometry assessment for the integrin CD103 [8, 9] with a mean of 91.23% of DP group positive, compared to 27.47% of the DN group (*p* = 0.0007 paired t test) (Fig. 3a).

### Functional assessment

To confirm the DP population maintains a functional phenotype, lymphocyte containing OAC single cell suspensions were stimulated by incubation with PMA and Ionomycin. CD107a staining was higher at baseline in the DP group compared to the DN suggesting increased degranulation in the presence of autologous tumour alone (Fig. 3b) and there was a further increase in CD107a staining on stimulation, which while modest, indicates additional capacity for cytotoxicity.

Both populations appeared able to produce more IFNγ and TNFα on stimulation while Perforin and Granzyme B production was unchanged by this (Fig. 3c). These results point to a DP population with maintained ability to produce the cytolytic molecules Granzyme B and Perforin and capacity to increase degranulation, IFNγ and TNFα production on stimulation, in keeping with a functional phenotype.

## Discussion

A high degree of tumour infiltration by CD8+ T lymphocytes is associated with improved survival following resection of oesophageal adenocarcinomas, though the impact made by the composition of these TILs remains poorly understood. Here we demonstrate that OACs are often highly infiltrated by a population of PD-1 and CD39 positive, antigen experienced CD8+ T lymphocytes. Detailed characterisation of this population reveals lymphocytes with modest expression of TIM3 and LAG3, and high expression of the canonical TRM marker CD103.

It can be speculated that this population is enriched for cancer specific lymphocytes given their expression of CD39 and CD103 alongside often high levels of PD-1, as well as the increased transcription of CXCL13 [10, 13, 19]. They too have transcriptional signatures in keeping with proliferation and TCR signalling, produce cytolytic effector molecules at or near their capacity, and can increase their secretion of IFNγ and TNFα on stimulation. That this profile would also fit for a TRM population is strengthened by the observation of enrichment for TRM gene sets, as well as Reactome signatures for IL-10 signalling, which while often inhibitory to CD8+ effector cells, has a role in TRM development [20].

We demonstrate that PD-1+ CD39+ TIL proportion correlates with improved clinical outcome in OAC after surgery, and that this may be impacted by prior Chemotherapy and CRT in some individuals, though this would need further evaluation in a larger cohort. This is of importance given the evolving interest in the use of ICB in the perioperative setting, including the observation of high pathological complete response rates in recent phase 2 studies utilising a run-in phase of such therapies alone before combination with traditional chemotherapy approaches [21]. The data presented here provides additional data on the PD-1, CXCL13 and CD103+ CD8+ OAC TILs that correlate with ICB response in these studies, and the impact that current standard of care chemotherapy and chemoradiotherapy may have upon their abundance. Considerations that are crucial when designing optimal perioperative approaches for such a difficult to treat cancer.

## Supplementary Information

Below is the link to the electronic supplementary material.

Supplementary file1 (XLSX 121 kb)

Supplementary file2 (PDF 287 kb)

## Data Availability

The datasets generated during the current study are available from the corresponding author on reasonable request.
