# Supplementary material for: High proportion of PD-1 and CD39 positive CD8+ tissue resident T lymphocytes correlates with better clinical outcome in resected human oesophageal adenocarcinoma
Source: Cancer Immunol Immunother. 2024 Sep 5;73(11):213. doi: 10.1007/s00262-024-03799-y (PMC11377377; doi:10.1007/s00262-024-03799-y)
Supplement: Supplementary file 2 — Supplementary file2 (PDF 287 kb) [file 262_2024_3799_MOESM2_ESM.pdf]

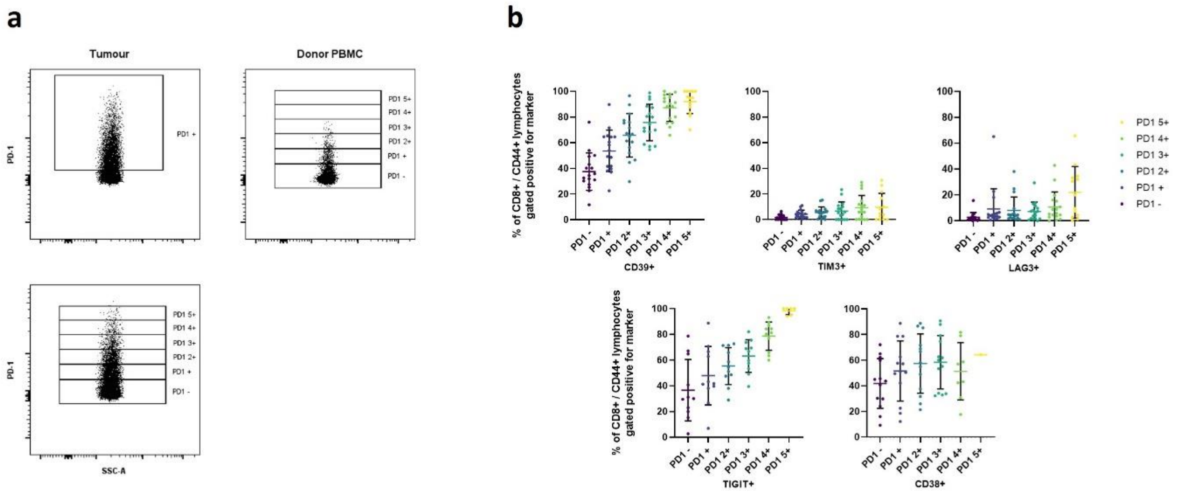

Fig. S1 Exhaustion and activation marker positivity by degree of PD-1 positivity. (**a**) Gating strategy for assessment of antigen experienced CD8+ TILs degree of PD-1 positivity, and comparison with PBMC from healthy donor. (**b**) Percentage of antigen experienced CD8+ TILs positive for CD39, TIM3, LAG3, TIGIT and CD38 by degree of PD-1 positivity as defined by the gating strategy in A.

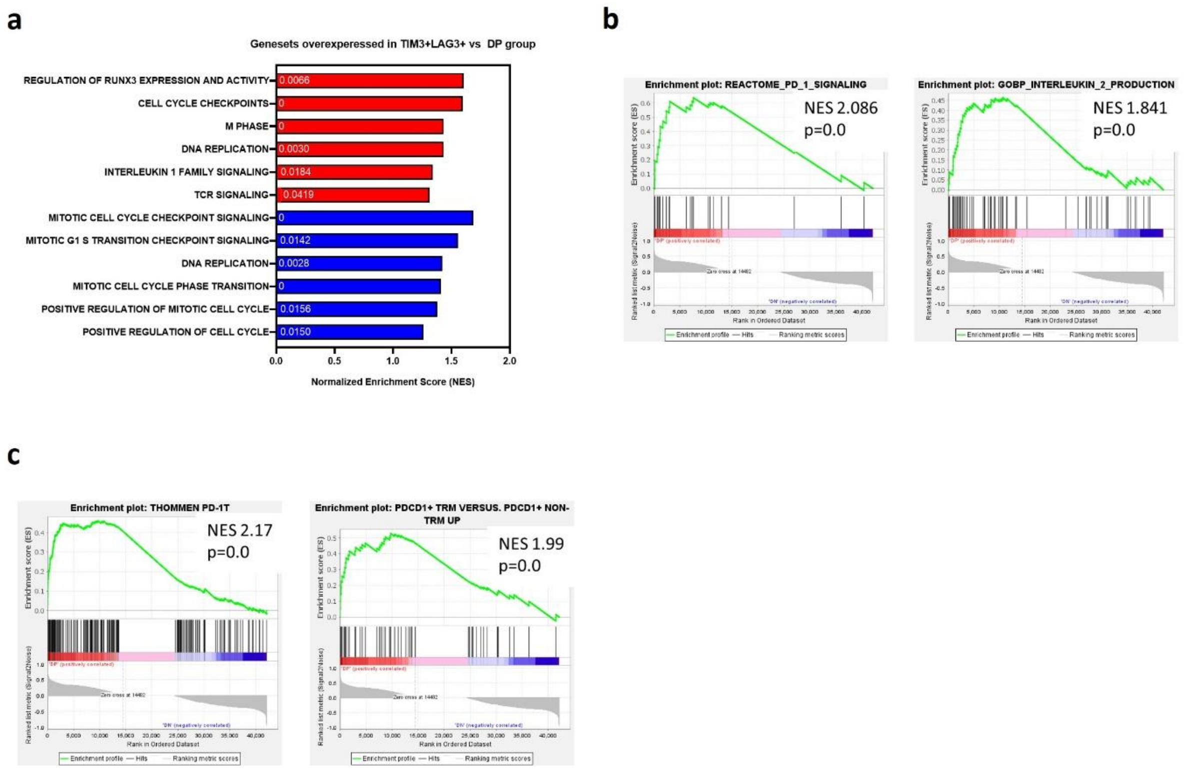

Fig. S2 Transcriptomic assessment of PD-1 and CD39 positive populations of antigen experienced CD8+ TILs. (**a**) Normalised enrichment scores (NES) for Reactome (red) and Gene Ontology (blue) signatures in TIM3+LAG3+ vs DP populations, as assessed by gene set enrichment analysis (GSEA). P values for each gene set labelled within the bar in white. (**b-c**) Exemplar gene set enrichment plot for key Reactome and GO gene sets (**b**) as well as for gene sets from previously published works (**c**). NES and p value included.
